# Prevalence of orthorexia nervosa: a systematic review and meta-analysis protocol

**DOI:** 10.1136/bmjopen-2024-096802

**Published:** 2025-05-23

**Authors:** Tayla Eckley, Shae E Quirk, Julie A Pasco, Mariel Messer, Lana J Williams

**Affiliations:** 1 Institute for Mental and Physical Health and Clinical Translation, Deakin University, Geelong, Victoria, Australia; 2 University Hospital, Geelong, Victoria, Australia; 3 Department of Medicine-Western Health, The University of Melbourne, St Albans, Victoria, Australia; 4 Department of Epidemiology and Preventative Medicine, Monash University, Melbourne, Victoria, Australia; 5 School of Psychology, Deakin University, Geelong, Victoria, Australia

**Keywords:** Eating disorders, MENTAL HEALTH, Systematic Review, Prevalence

## Abstract

**Abstract:**

**Introduction:**

Orthorexia nervosa (ON) is a newly recognised condition characterised by an excessive fixation with healthy eating, yet the prevalence of ON is poorly understood. This protocol presents the methodology to undertake a systematic review and meta-analysis on the prevalence of ON in a wide range of populations (including general population and ‘high-risk’). To the authors’ knowledge, the proposed review will be the first systematic review to critically appraise the quality and quantity of evidence on this topic.

**Methods:**

The protocol has been developed following the Preferred Reporting Items for Systematic Reviews and Meta-Analyses Protocols guidelines. Eligible studies will be identified through a systematic search of electronic databases (eg, Medline Complete, PsycInfo and CINAHL complete via the EBSCOHost platform and Embase). Two reviewers will independently screen and review the full text of records, extract the data and critically appraise the evidence using the Joanna Briggs Institute critical appraisal checklist for prevalence studies. A descriptive synthesis will present the characteristics of the included studies and key findings in text and tables. Where appropriate, meta-analysis will be conducted to determine the proportion of individuals with ON (yes/no) according to population groups of interest (ie, general and ‘high-risk’ populations) and/or ON tools.

**Ethics and dissemination:**

This review will include published data only; thus, ethical permission will not be necessary. Results of this review will be published in a relevant peer-reviewed scientific journal and presented at conferences in related fields.

**PROSPERO registration number:**

CRD42024576557.

**STRENGTHS AND LIMITATIONS OF THIS STUDY**

- The proposed review will provide a thorough synthesis of the existing data on the prevalence of orthorexia nervosa derived from a range of different self-report measurement tools, which is yet to be undertaken.
- Participants in eligible studies will have been recruited from either general or ‘high-risk’ population settings, allowing for a detailed synthesis of the prevalence of orthorexia in different population groups.
- Potential limitations include heterogeneity of the existing evidence and varying methodological quality of included studies.

## Introduction

 Orthorexia nervosa (ON) is an emerging psychological concern characterised by a pervasive preoccupation with, and the consumption of, a healthy diet.^1^,^3^ These cognitions and eating behaviours may initially manifest as benign efforts to avoid unhealthy foods; however, they can develop into a rigid pattern of thinking and disordered eating behaviours characterised by an excessive fixation on the perceived quality of foods and restricted eating.^3 4^ To date, ON has not been classified as a mental disorder according to the Diagnostic and Statistical Manual of Mental Disorders, Fifth Edition or the International Classification of Diseases, Eleventh Revision^5^,^7^; and thus, epidemiological research on the nature and prevalence of ON is still in its infancy.

However, proposed diagnostic criteria include obsessive behaviours and preoccupation with a healthy diet, emotional distress resulting from a lapse in self-imposed dietary rules, and physical and psychosocial impairment resulting from adherence to rigid dietary patterns.^8^ Other symptoms of ON described in the literature involve viewing food primarily as a source of health rather than pleasure, distress when in proximity to unhealthy foods, persistent belief that dietary practices are healthy despite indications of malnutrition, moral judgement of others based on dietary habits and body image distortions related to a sense of physical impurity.^9 10^

Several measurement tools have been developed to measure ON, with the Bratman Orthorexia Test (BOT) being one of the first to emerge as an informal screening tool for ON traits.^11^ However, evidence has shown the BOT to lack basic psychometric properties, including poor validity and reliability of the items, as well as not including a guideline for the interpretation of scores.^9 12 13^ Despite this, the BOT became the basis for the most widely known and most commonly used measurement of ON, the ORTO-15 and its variants (ie, ORTO-9, ORTO-11).^14^ However, the ORTO-15 too has been criticised for its poor psychometric properties. In particular, concerns have been raised regarding its poor internal consistency, low content validity and limitations regarding the scoring scheme and interpretation of scores.^15^,^17^ Reportedly, the BOT and ORTO-15 both inadequately address the obsessive-compulsive traits characteristic of ON. Thus, these scales also lack the ability to differentiate between ON and general health-conscious eating behaviours, leading to a possible overestimation of the prevalence of ON.^18^ There has been in-depth scientific discourse regarding the methods and approaches to measuring ON, with a particular focus on the limitations of the ORTO-15. Some researchers have argued that data obtained from the ORTO measures are invalid due to the aforementioned psychometric limitations of this measure^19^; meanwhile, others point out that the data obtained from these measures form a critical stepping stone in the development of more psychometrically sound measurement tools, as well as the progress towards a more homogeneous definition of ON.^20^ Several newer measurement tools have since been developed and have, to date, demonstrated adequate psychometrics. Newer tools include the Eating Habits Questionnaire (EHQ),^21^ Dusseldorf Orthorexia Scale (DOS),^22^ Teruel Orthorexia Scale (TOS),^15^ Orthorexia Nervosa Scale (ONS)^23^ and Orthorexia Nervosa Inventory (ONI).^8^ Each of these scales has demonstrated adequate internal consistency, test–retest reliability and has been validated in numerous languages and populations.^13 17^ To date, the tools are exclusively self-reported. Thus, it is possible that the use of different tools could yield varying prevalence estimates of ON, which are yet to be thoroughly compared.

The large number of tools that exist to measure ON might be attributed to the difficulty conceptualising ON as a distinct mental disorder. There has been ongoing debate as to whether ON should be classified as a distinct eating disorder or variant of one of the existing eating disorders. Arguments for the former suggest that the underlying motivations of ON differ from those of existing eating disorders such as anorexia nervosa and bulimia nervosa. In contrast to anorexia nervosa and bulimia nervosa, people with ON tend to be preoccupied with the quality of what they consume, rather than the quantity,^4 24^ and dietary restrictions do not appear to be as driven by excessive fear of weight gain or distorted body image.^3^ Whereas arguments that ON is a variant of existing eating disorders suggest that, similar to anorexia nervosa and bulimia nervosa, ON involves an intense fear of consuming unwanted foods.^3^ In particular, the rigid dietary rules and restrictive behaviour observed in ON appear to be similar to behaviours exhibited in anorexia nervosa. Like anorexia, ON is associated with a genetic predisposition to perfectionism, desire for control and poor insight into the consequences of their dietary behaviours.^2 3 24^

The differing views on approaches to measuring and classifying ON have likely been a barrier to determining the epidemiology of ON. For example, the prevalence of ON in existing studies appears to vary widely from 7% in a convenience sample aged over 16 years (n=404) recruited from the Institute of Food Sciences, University of Rome ‘La Sapienza’ in Italy^25^ to 82% among a subsample of Opera singers (n=36 ON/44) in the Turkish State Opera and Ballet and the Bilkent University Symphony Orchestra (n=53 ON/94 total sample)—both studies using the ORTO-15^26^ to measure ON. Moreover, several studies that report higher prevalence of ON are among samples of participants considered to be ‘high-risk’, including adolescents, university students, healthcare professionals, dieticians, athletes and performance artists.^4 24^ These particular groups are considered to have greater susceptibility to ON traits due to the focus on perceived attainment of optimal health in their occupation/profession (eg, medical professionals and dieticians), fitness and physical performance (eg, athletes) and in some cases physical appearance (eg, performance artists such as dancers).^4^ Additionally, for adolescents, the increased susceptibility to ON may be attributed to a developing sense of independence and individual responsibility in various aspects of their day-to-day lives.^24^ However, the occurrence of ON in population-based studies that are more broadly representative of the general population worldwide is not definitively known, nor is it well understood which population groups have comparatively higher occurrences of ON than others. As data regarding the prevalence and characteristics of ON outside of these high-risk populations are limited, it remains difficult to conceptualise ON as a disorder. Thus, this information is crucial to aid in the classification of the condition.

The authors conducted a preliminary search in Medline Complete and PROSPERO on 17 June 2024, which revealed existing reviews on this topic. First, Strahler presented a systematic review and meta-analysis of the sex differences in prevalence estimates ON according to four different measurement tools (BOT, EHQ, DOS and ORTO-15) in 67 studies.^27^ Inconsistent findings were reported concerning sex differences in the prevalence of ON, which appeared to be due to the tools used to measure ON; for example, no sex differences were found using the most common measure of ON, the ORTO-15.^27^ Meanwhile, limitations of the review were reported to include varying cut points to identify ON across the studies.

Separately, Hafstad and colleagues examined the prevalence of ON among exercising populations specifically.^28^ The key findings of this review indicated that the pooled prevalence of ON is 55.3% among exercising populations according to the ORTO-15 and DOS.^28^ However, it was noted that the use of the ORTO-15 yielded higher prevalence estimates, while the DOS appeared to yield lower estimates. Limitations were also noted to include that the ORTO-15 was the most used measure to identify ON, which the authors postulated may inflate prevalence estimates in the study populations.^28^

Recently, López-Gil and colleagues undertook a comprehensive systematic review and meta-analysis on the prevalence of ON among 30 476 individuals from 18 countries without a co-occurring eating disorder or other mental and physical health conditions, and chiefly used the ORTO-15 questionnaire to measure ON.^29^ The overall pooled prevalence of ON was reported to be 27.5% with no significant sex differences observed. Additionally, the highest proportion of ON was found among people focused on sports performance or body composition (34.5%), which supports the notion that certain groups may be more susceptible to the development of ON.^29^ Importantly, the authors aimed to overcome prior limitations of the existing literature by defining cut-points for determining ON; these included the original cut-point developed for the ORTO-15 (scores less than 40) and a more conservative cut-point (scores less than 35) developed to account for the potential inflation of ON prevalence estimates.^29^

Other key findings included that neither body mass index (BMI) nor age was associated with the proportion of ON symptoms.^29^ A key limitation of the studies included in the review pertained to the quality of the evidence; it was identified that the main sources of bias were related to the lack of representativeness of the samples.^29^ Thus, including a wider range of measurement tools to identify ON might yield a wider range of eligible studies, including studies with more representative study designs.

While these reviews significantly contribute to the knowledge in the field of ON research, there are some key gaps that are yet to be filled. For example, the pooled prevalence of ON, according to different population groups of interest, as well as using the full range of eligible measurement tools, is yet to be undertaken in one review. In addition, the authors will aim to include relevant published peer-reviewed grey literature (ie, thesis and dissertations). Furthermore, factors that might influence the prevalence of ON, including study characteristics, pertinent sociodemographic characteristics and health risk factors are yet to be thoroughly explored.

This protocol presents the methodology to undertake a systematic review and meta-analysis on the prevalence of ON. Specifically, the research questions guiding the proposed review are:

1. What is the prevalence of ON in representative population-based samples?
2. What is the prevalence of ON in specific population groups of interest including ‘high-risk’ groups?
3. For the questions above, does the prevalence of ON differ according to study characteristics (eg, study design, ON measurement tool, sample size and critical appraisal scores), pertinent sociodemographic characteristics (eg, age, sex, socio-economic status, occupation characteristics, health risk factors (eg, BMI, physical activity, smoking) and mental health status (eg, presence of eating disorders or other mental disorders)?

## Methods and analysis

This protocol has been developed following the Preferred Reporting Items for Systematic Reviews and Meta-Analyses (PRISMA) Protocols guidelines.^30 31^

### Eligibility criteria

The eligibility criteria for the proposed review are described in the following sections according to eligible populations, phenomena of interest and study designs.

### Population

Eligible samples include population-based adolescents and/or adults (aged 13+ years) of any sex, gender, nationality, ethnicity, race or culture (eg, in the community). In addition, specific population groups of interest include those considered ‘high-risk’:

- Athletes (eg, athletics, martial arts, sportsperson).
- People focused on or adhering to a specific diet/dietary pattern (vegan, vegetarian).
- People with mental health conditions (eg, eating disorders, obsessive compulsive disorder).
- Performing artists (eg, ballet dancers, singers).
- High school and university students (eg, students studying medicine, nutrition, dietetics, exercise and sport science).
- Workers in healthcare occupations/professions (eg, medical doctors, nurses, dieticians).

### Phenomena of interest

The phenomena of interest is ON characterised as rigid patterns of thinking and eating behaviours involving an obsessive fixation on the perceived quality of food and identified by an existing self-report tool. Eligible self-report tools for the measurement of ON include:

- BOT
- ORTO-15 (other variants of the ORTO such as ORTO-11, and ORTO-R are also considered eligible)
- EHQ
- DOS
- TOS
- ONS
- ONI

### Study designs

Given the focus on prevalence studies, eligible study designs will include descriptive cross-sectional studies/prevalence surveys or cohort studies. Prevalence studies derived from a specific time point in a randomised controlled trial or case–control design may be considered. Publication types involving editorials, commentaries, case reports and conference abstracts are ineligible.

Studies published in any language will be considered. Studies will be restricted to those published since 1997 as the phenomenon of interest had not been described as ON prior to this date.

### Search strategy

Eligible studies will be identified through a search of electronic databases in the fields of medicine, health and psychology (eg, Medline Complete, PyscInfo and CINAHL Complete via the EbscoHost platform and Embase). A preliminary search for Medline Complete via the EBSCOhost platform has been developed using a Medical Subject Headings (MeSH) and keywords analyser tool, and from mapping these in a document, which was based on existing relevant literature. The MeSH and keywords identified were searched line by line (in title and abstract fields), and then combined using Boolean Operators. The preliminary search will be refined in consultation with a Librarian and translated for the other databases. The evidence sources considered relevant are peer-reviewed journal articles. Grey literature, such as published dissertations, will be searched using an adapted search for ProQuest Dissertations & Theses Global. The preliminary search was conducted on 28 July 2024; the results are presented in online supplemental table 1. There will be no restrictions on the date of publication.

### Study selection

Records identified from the search strategy will be exported into EndNote V.X9 reference management software, where duplicates will be removed. Remaining references will be exported into Covidence, an online software used for systematic review data management.^32^ Prior to screening, the eligibility and exclusion criteria will be pilot tested by two reviewers by screening a randomly selected sample of the identified records (n=15 records) for both the screening and full-text review stages. Acceptable agreement for the pilot tests will be defined as fewer than 5% conflicts between the independent screeners. If there is a higher rate of conflicts, the review team will discuss potential issues and make any necessary modifications to the eligibility criteria.

Following the pilot, titles and abstracts of identified records will be independently screened by two reviewers. Full-text articles will be retrieved for the records that satisfy the eligibility criteria in the title and abstract screening phase. The full-text review of articles will be undertaken independently by the same two reviewers. Any potential conflicts will be resolved by a third reviewer at the screening and full-text stages. Finally, the reference lists of eligible studies will also be exported using Scopus and screened to identify any further potential relevant records.

### Data management and extraction

Data will be extracted from eligible studies by two independent reviewers using a custom data extraction tool (see online supplemental table 2). The data extracted will include specific details about the participants/populations (eg, sample size, age, population group of interest and other relevant sociodemographic factors), study methods (eg, aims, study design, recruitment, statistical analyses), phenomena of interest (definition of orthorexia, tool and scoring) and results of relevance to the review question(s), including descriptive statistics (ie, frequencies and percentage with CIs/SE) and/or other relevant statistics for reporting scale scores (ie, mean and SD) will be extracted. The final data extraction tool will be informed by a consultation with a statistician and will be piloted by two reviewers prior to commencement. Potential conflicts will be resolved by discussion with a statistician and/or supervising author. Authors of papers may be contacted to request missing/additional data, if relevant.

### Critical appraisal of included studies

Eligible articles will undergo critical appraisal by two reviewers independently using the Joanna Briggs Institute (JBI) checklist for prevalence studies.^33^ The JBI critical appraisal checklist for studies reporting on prevalence data has been selected due to the appropriateness for assessing the eligible study designs. This checklist will be used to assess numerous aspects of eligible articles, including the use of appropriate sampling strategies, valid measurement of the phenomenon of interest, appropriate statistical analysis and adequate response rates.^33^ Potential conflicts will be resolved by discussion between the two reviewers and/or supervising author. All studies, regardless of the results of the critical appraisal, will undergo data extraction.

### Data synthesis and presenting and reporting results

The presentation and reporting of the results will adhere to the PRISMA guidelines.

A PRSIMA flow diagram will be used to document the screening and selection process, including reasons for exclusion at the full-test stage. The characteristics of the studies will be presented in text and tables. A descriptive synthesis of the key findings will also be presented in text and visually. The synthesis will involve the presentation of the prevalence of ON according to the population groups of interest and ON tool, where possible. The discussion will address the research questions, including a discussion on potential variation of prevalence estimates due to any identified population (eg, age, sex, gender, socio-economic status, occupation/profession characteristics) or study characteristics (eg, study design, ON measurement tool, sample size and critical appraisal scores). If appropriate, the strength and quality of the body of evidence will be determined using modified criteria for prevalence studies. Regardless of critical appraisal scores, the results of all studies will be included in the descriptive synthesis.

### Meta-analysis

Where appropriate, one-sample binary data meta-analysis will be undertaken to determine the proportion of individuals with ON (yes/no)—according to the population groups of interest and/or ON tools. The meta esize command will be used to compute the Freeman–Tukey double-arcsine-transformed proportion for each eligible study. The results will be reported as proportions (%) with 95% CIs and presented graphically in forest plots. Subgroup analyses may also be undertaken further to explore the proportion of ON according to population and study characteristics. Possible publication bias will be investigated by visually inspecting funnel plots. Further details regarding the analyses will be presented in the ensuing review.

## Discussion

The proposed systematic review will provide a comprehensive synthesis of the prevalence of ON in general populations worldwide and in a range of specific population groups of interest. Additionally, the proposed review will synthesise the prevalence of ON according to a broad range of existing self-report measurement tools. Thus, this review will inform an ongoing discussion in the literature concerning current approaches to defining and measuring ON. Furthermore, through critical appraisal of studies, this review will identify opportunities to comment on the quality of evidence produced on this topic and make recommendations for future research. In terms of possible limitations, there is potential for heterogeneity of the existing evidence and varying methodological quality of included studies. For example, the pooled analysis will be dependent on the availability of comparable study designs and methods used to assess the presence of ON.

## Ethics and dissemination

This systematic review will include published data only and therefore ethical permission will not be required. However, ethical and governance standards will be abided by, in respect to data management, presentation and dissemination of results. Results of this review will be presented in a related peer-reviewed scientific journal as well as through presentations at conferences related to mental health. The review is aimed to be completed by December 2025.

## Supplementary material

10.1136/bmjopen-2024-096802 online supplemental file 1
