# Supplementary material for: Prevalence of orthorexia nervosa: a systematic review and meta-analysis protocol
Source: BMJ Open. 2025 May 23;15(5):e096802. doi: 10.1136/bmjopen-2024-096802 (PMC12104933; doi:10.1136/bmjopen-2024-096802)
Supplement: online supplemental file 1 [file bmjopen-15-5-s001.pdf]

Supplementary Table 1: Preliminary search strategy developed for Medline Complete via the EbscoHost platform

S37	S33 AND S36	2,351
S36	S34 OR S35	2,293,211
S35	S28 OR S29 OR S30 OR S31 OR S32	1,101,588
S34	S24 OR S25 OR S26 OR S27	1,324,966
S33	S1 OR S2 OR S3 OR S4 OR S5 OR S6 OR S7 OR S8 OR S9 OR S10 OR S11 OR S12 OR S13 OR S14 OR S15 OR S16 OR S17 OR S18 OR S19 OR S20 OR S21 OR S22 OR S23	9,511
S32	(AB incidences)	941,426
S31	(TI incidences)	127,806
S30	(AB incidence)	941,426
S29	(TI incidence)	127,806
S28	(MH “Incidence”)	308,929
S27	(AB prevalence)	812,534
S26	(TI prevalence)	180,343
S25	(MH “Cross-Sectional Studies)	508452
S24	(MH “Prevalence”)	355,546
S23	(AB “Orthorexia Nervosa Inventory”)	11
S22	(TI “Orthorexia Nervosa Inventory”)	5
S21	(AB “Barcelona Orthorexia Scale”)	3
S20	(TI “Barcelona Orthorexia Scale”)	2
S19	(AB “Teruel Orthorexia Scale”)	32
S18	(TI “Teruel Orthorexia Scale”)	8
S17	(AB “Eating Habits Questionnaire”)	82
S16	(TI “Eating Habits Questionnaire”)	8
S15	(AB “ORTO”)	85
S14	(TI “ORTO”)	15
S13	(AB "Düsseldorf Orthorexia Scale")	24
S12	(TI "Düsseldorf Orthorexia Scale")	3
S11	(AB “orthorexia self-test”)	3
S10	(TI “orthorexia self-test”)	1
S9	AB ((orthorexia) N2 (scale* or questionnaire* or survey* or	113

	inventory or test* or “self report*”))	
S8	TI ((orthorexia) N2 (scale* or questionnaire* or survey* or inventory or test* or “self report*”))	31
S7	(AB orthorexi*)	448
S6	(TI orthorexi*)	411
S5	(MH “Orthorexia Nervosa”)	125
S4	(AB preoccupation) N3 (AB "healthy eating")	26
S3	(TI preoccupation) N3 (TI "healthy eating")	3
S2	(MH “Diet, Healthy”)	7,359
S1	(MH “Obsessive Behavior+”)	1676

Supplementary Table 2: Preliminary data extraction form

Study characteristics					Population characteristics			Measurement of ON			Results		
Citation	Year	Country/ region	Aims	Recruitment approach	Population group<br>• Population-based, 'high risk' group	Sample size (subgroups)	Sample characteristics<br>• Age, sex, gender, socioeconomic status, occupation characteristics	Tool	Scoring	Definition of ON	Analyses	Percentage (%) ON<br>• Total group, subgroup(s), and by other relevant sample characteristics	M (SD)
