# Supplementary material for: Prevalence of orthorexia nervosa: a systematic review and meta-analysis protocol
Source: BMJ Open. 2025 May 23;15(5):e096802. doi: 10.1136/bmjopen-2024-096802 (PMC12104933; doi:10.1136/bmjopen-2024-096802)
Supplement: Supplementary file 2 [file bmjopen-2024-096802.reviewer_comments.pdf]

PEER REVIEW HISTORY

BMJ Open publishes all reviews undertaken for accepted manuscripts. Reviewers are asked to complete a checklist review form and are provided with free text boxes to elaborate on their assessment. These free text comments are reproduced below.

ARTICLE DETAILS

Title (Provisional)

The prevalence of orthorexia nervosa: A systematic review and meta-analysis protocol

Authors

Eckley, Tayla; Quirk, Shae; Pasco, J; Messer , Mariel; Williams, Lana

---

VERSION 1 - REVIEW

**Reviewer**	**1**
**Name**	**López-Gil, José**
**Affiliation**	**Universidad de Las Américas Facultad de Ciencias de la Salud**
**Date**	**22-Feb-2025**
**COI**	**None**

Thank you for the opportunity to review the manuscript titled “The prevalence of orthorexia nervosa: A systematic review protocol”. While the topic is relevant, I regret to inform you that I cannot recommend its acceptance for publication in its current form.

A recent and comprehensive meta-analysis by López-Gil et al. (doi: 10.7189/jogh.13.04087) has already synthesized the available evidence on this subject, addressing key aspects such as prevalence trends, sex differences, and populations at higher risk for orthorexia nervosa. However, the authors of the present manuscript have neither cited nor engaged with this important work, which is a significant omission.

Additionally, recent discussions in the scientific community, including letters to the editor (Barrada & Meule, doi: 10.7189/jogh.14.03007; López-Gil et al., doi: 10.7189/jogh.14.03053), have critically examined the methodology commonly used in orthorexia research, particularly the limitations of the ORTO-15 questionnaire. These debates highlight the need for careful interpretation of prevalence estimates and the reconsideration of cutoff points for classification. Given these ongoing discussions, it is concerning that the authors have not acknowledged or addressed these relevant contributions.

Since the current study does not introduce substantially new findings, methodological advancements, or theoretical insights beyond what has already been extensively reviewed, its added value to the literature appears to be limited.

I appreciate the opportunity to review this work and hope these comments assist in the editorial decision-making process.

Reviewer **2**

Name **Hammerle, Florian**

Affiliation **Universitätsmedizin Mainz, Klinik und Poliklinik für Kinder- und Jugendpsychiatrie und -psychotherapie**

Date **01-Apr-2025**

COI **None**

Thank you very much for the opportunity to review the manuscript “The prevalence of orthorexia nervosa: A systematic review protocol”.

The topic of ON seems relevant and a systematic review, perchance complemented with a meta analysis, is an interesting methodological approach in this field. The manuscript is well written and in most parts good to understand.

Nevertheless, there are some issues which should be addressed:

The pivot point is ON and definitions of ON. In the methods (line 166 ff.) ON is characterized as “rigid patterns of thinking and eating behaviours involving an obsessive fixation on the perceived quality of food and identified by an existing self report tool”. This definition is very vague. Although I understand that there is a large heterogeneity in definitions maybe the definition could be sharpened (e.g. by including a relevant burden of diseass, functional impairment or a duration of symptoms). Perhaps there is a convergence in regard to these aspects between all relevant instruments. Further on, it is unclear, if the authors want to include studies using the BOT and it’s derivats (ORTO-..-), which are often used but are described as unreliable and not very valid. It could be a solution to describe all eligible questionnaires listwise.

Another major issue is the compilation of groups considered associated with high risk: it is unclear why the authors choose some specific groups. Either the introduction should be augmented with more literature depicting all groups with a higher risk of ON and/or the hypothesis section should be used to explain why some groups could possibly be associated with a higher risk of ON.

There are two intended levels of analysis: general population and high-risk groups. In some parts of the manuscript (e.g. Abstract / Conclusion) you use the sequence high-risk – general population whereas other parts (e.g. Introduction / line 108) you describe prevalences in general population (“convenience sample”) before reporting on high-risk groups. Please use the same sequence and the same key term “general population” throughout the whole manuscript.

I go on to describe other issues with direct reference to the passages in the text:

Abstract: method: the two different levels of analysis (general population/high risk groups) should be included.

Introduction: "Other traits of ON" (line 69): it's more like symptoms rather than traits.

Methods: eligible samples include participants from 14+ years. Why did you use 14 years as cut-off for adolescence? Please describe this further in the manuscript.

Just a minor issue: the enumeration of athletics, martial arts, sport (line 160) seems not coherent. "Sport" is the umbrella term whereas martial arts and athletics are types of sports.

The authors state, that they will not use a restriction of date (line 177). When considering risk groups as "eating disorders" and/or "obsessive compulsive disorders" different diagnostic systems (e.g. DSM-III/DSM-IV and DSM-5) conceptualize mental disorders different. Therefore, e.g. studies from around 1980 (although it could be expected that there are very few studies analyzing ON) are not comparable to studies from around 2020. It could be useful to focus on a specific time frame.

Study selection:

Just a minor issue: line 196: I expect that you agreed upon the sample of studies you want to screen beforehand. If so, please specify exactly the number of studies (15) without e.g.

Conclusions:

Implications and Strengths/Limitations are missing. Please add this to the manuscript.

VERSION 1 - AUTHOR RESPONSE

Reviewer: 1
Dr. José López-Gil, Universidad de Las Américas Facultad de Ciencias de la Salud
Comments to the Author:

1. ***Thank you for the opportunity to review the manuscript titled "The prevalence of orthorexia nervosa: A systematic review protocol". While the topic is relevant, I regret to inform you that I cannot recommend its acceptance for publication in its current form.***

A recent and comprehensive meta-analysis by López-Gil et al. (doi: 10.7189/jogh.13.04087) has already synthesized the available evidence on this subject, addressing key aspects such as prevalence trends, sex differences, and populations at higher risk for orthorexia nervosa. However, the authors of the present manuscript have neither cited nor engaged with this important work, which is a significant omission.

Author response. Thank you for reviewing our protocol and the constructive feedback. The authors now acknowledge this important prior work and the significant contribution it has made to the literature. Please refer to our response to the editor's comment (number six).

2. ***Additionally, recent discussions in the scientific community, including letters to the editor (Barrada & Meule, doi: 10.7189/jogh.14.03007; López-Gil et al., doi: 10.7189/jogh.14.03053), have critically examined the methodology commonly used in orthorexia research, particularly the limitations of the ORTO-15 questionnaire. These debates highlight the need for careful interpretation of prevalence estimates and the***

reconsideration of cutoff points for classification. Given these ongoing discussions, it is concerning that the authors have not acknowledged or addressed these relevant contributions.

Author response. Please see additional text in the introduction to acknowledge this point as follows:

- *Lines 91-96.*

There has been in depth scientific discourse regarding the methods and approaches to measuring ON, with a particular focus on limitations of the ORTO-15. Some researchers have argued that data obtained from the ORTO measures are invalid[19], meanwhile others point out the data obtained from these measures forms a critical stepping stone in the development of more psychometrically sound measurement tools as well as the progress towards a more homogenous definition of ON[20].

- *Lines 160-164.*

Importantly, the author's aimed to overcome prior limitations of the existing literature, by defining cut-points for determining ON; these included the original cut- point developed for the ORTO-15 (scores less than 40) and a more conservative cut- point (scores less than 35) developed to account for the potential inflation of ON prevalence estimates[29].

3. ***Since the current study does not introduce substantially new findings, methodological advancements, or theoretical insights beyond what has already been extensively reviewed, its added value to the literature appears to be limited.***

I appreciate the opportunity to review this work and hope these comments assist in the editorial decision-making process.

Author response. Thank you for reviewing our manuscript, and again, providing constructive criticism. Please see revisions to our manuscript that highlight the added value of the current review:

- *Lines 171-180 (introduction)*

While these reviews significantly contribute to the knowledge in the field of ON research, there are some key gaps that are yet to be filled. For example, the pooled prevalence of ON, according to different population groups of interest, as well as utilising the full range of eligible measurement tools, is yet to be undertaken in one review. In addition, the authors will aim to include relevant published peer-reviewed grey literature (i.e., thesis and dissertations). Furthermore, factors that might influence the prevalence of ON including study characteristics, pertinent sociodemographic characteristics, and health risk factors are yet to be thoroughly explored.

- Lines 187-196 (research questions)

1. What is the prevalence of ON in representative population-based samples of the community?
2. What is the prevalence of ON in specific population groups of interest including "high risk" groups?
3. For the questions above, does the prevalence of ON differ according to: study characteristics (e.g., study design, ON measurement tool, sample size, and critical appraisal scores), pertinent sociodemographic characteristics (e.g., age, sex, socioeconomic status, occupation characteristics, health risk factors (e.g., BMI, physical activity, smoking), and mental health status (e.g., presence of eating disorders or other mental disorders)?

Reviewer: 2
Mr. Florian Hammerle, Universitätsmedizin Mainz
Comments to the Author:

1. ***Thank you very much for the opportunity to review the manuscript "The prevalence of orthorexia nervosa: A systematic review protocol".***

The topic of ON seems relevant and a systematic review, perchance complemented with a meta analysis, is an interesting methodological approach in this field. The manuscript is well written and in most parts good to understand.

Author response. We thank the reviewer for their review of our protocol and opportunity to revise our manuscript. Please see specific responses to each of the reviewers comments below.

2. ***Nevertheless, there are some issues which should be addressed:***

The pivot point is ON and definitions of ON. In the methods (line 166 ff.) ON is characterized as "rigid patterns of thinking and eating behaviours involving an obsessive fixation on the perceived quality of food and identified by an existing self report tool". This definition is very vague. Although I understand that there is a large heterogeneity in definitions maybe the definition could be sharpened (e.g. by including a relevant burden of diseass, functional impairment or a duration of symptoms). Perhaps there is a convergence in regard to these aspects between all relevant instruments. Further on, it is unclear, if the authors want to include studies using the BOT and it's derivats (ORTO-..-), which are often used but are described as unreliable and not very valid. It could be a solution to describe all eligible questionnaires listwise.

Author response. The list of eligible tools is now presented as follows:

- *Lines 219-227 (2.1.2Phenomena of interest)*

Eligible self-report tools for the measurement of ON include:

- Bratman Orthorexia Test (BOT)
- ORTO-15 (other variants of the ORTO such as ORTO-11, and ORTO-R are also considered eligible)
- Eating Habits Questionnaire (EHQ)
- Dusseldorf Orthorexia Scale (DOS)
- Teruel Orthorexia Scale (TOS)
- Orthorexia Nervosa Scale (ONS)
- Orthorexia Nervosa Inventory (ONI)

3. ***Another major issue is the compilation of groups considered associated with high risk: it is unclear why the authors choose some specific groups. Either the introduction should be augmented with more literature depicting all groups with a higher risk of ON and/or the hypothesis section should be used to explain why some groups could possibly be associated with a higher risk of ON.***

Author response. The explanation of why the authors have considered specific high risk groups is presented on lines 123-131 as follows:

- Moreover, several studies that report higher prevalence of ON are among samples of participants considered to be 'high-risk' including adolescents, university students, healthcare professionals, dieticians, athletes, and performance artists[4, 24]. These particular groups are considered to have greater susceptibility to ON traits due to the focus on perceived attainment of optimal health in their occupation/profession (e.g.,

medical professionals and dieticians), fitness and physical performance (e.g., athletes), and in some cases physical appearance (e.g., performance artists such as dancers)[4]. Additionally, for adolescents, the increased susceptibility to ON may be attributed to a developing sense of independence and individual responsibility in various aspects of their day to day lives[24].

4. ***There are two intended levels of analysis: general population and high-risk groups. In some parts of the manuscript (e.g. Abstract / Conclusion) you use the sequence high-risk – general population whereas other parts (e.g. Introduction / line 108) you describe prevalences in general population ("convenience sample") before reporting on high-risk groups. Please use the same sequence and the same key term "general population" throughout the whole manuscript.***

Author response. Text has been amended throughout; see the following lines for examples:

- *Lines 26-28:*

This protocol presents the methodology to undertake a systematic review and meta- analysis on the prevalence of ON in a wide range of populations (including general population and 'high-risk').

- *Lines 37-39:*

Where appropriate, meta-analysis will be conducted to determine the proportion of individuals with ON (yes/no) according to population groups of interest (i.e., general and 'high-risk' populations) and/or ON tools.

- *Lines 58-59:*

Participants in eligible studies will have been recruited from either general or 'high- risk' population settings

5. ***I go on to describe other issues with direct reference to the passages in the text:***

Abstract: method: the two different levels of analysis (general population/high risk groups) should be included.

Author response. The 'Abstract: method' has been revised as follows:

Methods: The protocol has been developed following the Preferred Reporting Items for Systematic Reviews and Meta-Analyses (PRSIMA) Protocols guidelines. Eligible studies will be identified through a systematic search of electronic databases (e.g., Medline Complete, PsycInfo, and CINAHL complete via the EBSCOHost platform and Embase). Two reviewers will independently screen and review the full text of records, extract the data, and critically appraise the evidence using the Joanna Briggs Institute critical appraisal checklist for prevalence studies. A descriptive synthesis will present the characteristics of the included studies and key findings in text and tables. Where appropriate, meta-analysis will be conducted to determine the proportion of individuals with ON (yes/no) according to population groups of interest (i.e., general and 'high-risk' populations) and/or ON tools.

6. ***Introduction: "Other traits of ON" (line 69): it's more like symptoms rather than traits.***

Author response. Line 69 has now been revised to "Other symptoms of ON…"

7. ***Methods: eligible samples include participants from 14+ years. Why did you use 14 years as cut-off for adolescence? Please describe this further in the manuscript.***

Author response. During the development of this protocol the authors noted some inconsistency in the literature regarding the definition of the 'adolescent' age range, with some authors suggesting adolescents should be defined as age 14+ while others suggested 13+.

After careful consideration of your comment and the relevant literature, we have decided to adjust the age of our eligible sample for ease of interpretation as follows:

- Eligible samples include population-based adolescents and/or adults (aged 13+ years)

8. ***Just a minor issue: the enumeration of athletics, martial arts, sport (line 160) seems not coherent. "Sport" is the umbrella term whereas martial arts and athletics are types of sports.***

Author response. The text has been revised to read as follows:

- "Athletes (e.g., athletics, martial arts, sportsperson)".

9. ***The authors state, that they will not use a restriction of date (line 177). When considering risk groups as "eating disorders" and/or "obsessive compulsive disorders" different diagnostic systems (e.g. DSM-III/DSM-IV and DSM-5) conceptualize mental disorders different. Therefore, e.g. studies from around 1980 (although it could be expected that there are very few studies analyzing ON) are not comparable to studies from around 2020. It could be useful to focus on a specific time frame.***

Author response. The line describing the restriction of date has been revised on lines 236-238 to the following:

- Studies will be restricted to those published since 1997 as the phenomenon of interest had not been described as ON prior to this date.

10. ***Study selection:***

Just a minor issue: line 196: I expect that you agreed upon the sample of studies you want to screen beforehand. If so, please specify exactly the number of studies (15) without e.g.

Author response. The 'e.g.' has been removed from line 196.

11. ***Conclusions:***

Implications and Strengths/Limitations are missing. Please add this to the manuscript.

Author response. Please see revised text on lines 314-324. Note that the conclusion section was removed from the main text based on feedback from the Research Editor, the implications and strengths/limitations have now been added to a 'Discussion' section in the main text.

The revised text is as follows:

- **Discussion**

The proposed systematic review will provide a comprehensive synthesis of the prevalence of ON in general populations world-wide and in a range of specific population groups of interest. Additionally, the proposed review will synthesise the prevalence of ON according to a broad range of existing self-report measurement tools. Thus, this review will inform an ongoing discussion in the literature

concerning current approaches to defining and measuring ON. Furthermore, through critical appraisal of studies, this review will identify opportunities to comment on the quality of evidence produced on this topic and make recommendations for future research. In terms of possible limitations, there is potential for heterogeneity of the existing evidence and varying methodological quality of included studies. For example, the pooled analysis will be dependent on the availability of comparable study designs and methods used to assess the presence of ON.
